# Supplementary material for: The Hunchback temporal transcription factor determines interneuron molecular identity, morphology, and presynapse targeting in the Drosophila NB5–2 lineage
Source: bioRxiv. 2024 Oct 7:2024.10.07.616945. Preprint. [Version 1] doi: 10.1101/2024.10.07.616945 (PMC11482779; doi:10.1101/2024.10.07.616945)
Supplement: Supplement 1 [file NIHPP2024.10.07.616945v1-supplement-1.pdf]

bioRxiv preprint doi: https://doi.org/10.1101/2024.10.07.616945; this version posted October 7, 2024. The copyright holder for this preprint (which was not certified by peer review) is the author/funder, who has granted bioRxiv a license to display the preprint in perpetuity. It is made available under aCC-BY 4.0 International license.

Supplemental Figure 1. NB5-2 can be identified by its stereotyped location in early embryos. NB5-2 (yellow in schematic) was identified using the NB marker *dpn* (magenta) and the row 6/7 expressing gene, *engrailed* (*en*; green). NB5-2 was identified as the most medial dpn+/en-negative NB, anteriorly adjacent to the engrailed domain. NB5-2 shows Hb/Kr expression in early stage 9 embryos (top panels) and Pdm expression by early stage10 (bottom panels). Scale bar: 5 µm.

bioRxiv preprint doi: https://doi.org/10.1101/2024.10.07.616945; this version posted October 7, 2024. The copyright holder for this preprint (which was not certified by peer review) is the author/funder, who has granted bioRxiv a license to display the preprint in perpetuity. It is made available under aCC-BY 4.0 International license.

Supplemental Figure 2. Prolonged Hunchback expression in NB5-2 generates fewer progeny expressing late TTFs.

(A) Wildtype NB5-2>GFP (green; top panels) and NB5-2>Hb (bottom panels) progeny expressing Kr at stage 14. Anterior left, lateral view. Scale bar: 4 μm.

(B) Wildtype NB5-2>GFP (top panels) and NB5-2>Hb (bottom panels) progeny expressing Pdm at stage 14.

(C) Wildtype NB5-2>GFP (top panels) and NB5-2>Hb (bottom panels) progeny expressing Grh at stage 16-17.

bioRxiv preprint doi: https://doi.org/10.1101/2024.10.07.616945; this version posted October 7, 2024. The copyright holder for this preprint (which was not certified by peer review) is the author/funder, who has granted bioRxiv a license to display the preprint in perpetuity. It is made available under aCC-BY 4.0 International license.

(D) Quantification of wildtype NB5-2>GFP (WT; avg=2.75, n=12 hemisegments, 3 animals) and NB5-2>Hb (HbOE; avg=11.91, n=11 hemisegments, 4 animals) neurons expressing Hb and Kr (*p-value*<0.0001).

(E) Quantification of all wildtype NB5-2>GFP (avg=6.67, n=12 hemisegments, 3 animals) and NB5-2>Hb (avg=17.45, n=11 hemisegments, 4 animals) neurons expressing Kr (*p-value*<0.0001).

(F) Quantification of wildtype NB5-2>GFP (avg=2.25, n=12 hemisegments, 3 animals) and NB5-2>Hb (avg=8.27, n=11 hemisegments, 3 animals) neurons expressing Hb and Pdm (*p-value*<0.0001).

(G) Quantification of all wildtype NB5-2>GFP (avg=7.67, n=12 hemisegments, 3 animals) and NB5-2>Hb (avg=11.09, n=11 hemisegments, 3 animals) neurons expressing Pdm (*p-value*=0.0017).

(H) Quantification of all wildtype NB5-2>GFP (avg=2.42, n=12 hemisegments, 3 animals) and NB5-2>Hb (avg=1.00, n=12 hemisegments, 3 animals) neurons expressing Grh (*p-value*=0.0002).

bioRxiv preprint doi: https://doi.org/10.1101/2024.10.07.616945; this version posted October 7, 2024. The copyright holder for this preprint (which was not certified by peer review) is the author/funder, who has granted bioRxiv a license to display the preprint in perpetuity. It is made available under aCC-BY 4.0 International license.

Supplemental Figure 3. Prolonged Hunchback expression increases the number of NB5-2 progeny that express Nkx6.

(A) Wildtype NB5-2>GFP (WT; green) progeny co-expressing Hb and the early-born TF, Nkx6, at stage 17 (dotted magenta). Anterior left, lateral view. Scale bar: 4 µm.

(B) WT NB5-2>GFP progeny expressing Nkx6 (B'), at stage 17. Scale bar: 4 µm.

(C) NB5-2>Hb progeny expressing Nkx6 (C').

bioRxiv preprint doi: https://doi.org/10.1101/2024.10.07.616945; this version posted October 7, 2024. The copyright holder for this preprint (which was not certified by peer review) is the author/funder, who has granted bioRxiv a license to display the preprint in perpetuity. It is made available under aCC-BY 4.0 International license.

Supplemental Figure 4. NB5-2 late-born neurons do not possess a diagonal projecting morphology.

TEM reconstruction of wildtype NB5-2 late-born neurons organized by dorsal commissural projections (left column) and ventral commissural projections (right column). Dorsal up, posterior view (left panel); Anterior up, ventral view (right panel).

bioRxiv preprint doi: https://doi.org/10.1101/2024.10.07.616945; this version posted October 7, 2024. The copyright holder for this preprint (which was not certified by peer review) is the author/funder, who has granted bioRxiv a license to display the preprint in perpetuity. It is made available under aCC-BY 4.0 International license.

Supplemental Figure 5. Idun1 expresses GABA neurotransmitter. TEM reconstruction of Idun1 (upper panels) and single labeled Hb+ NB5-2 neuron genetically labeled with membrane-bound epitope tag, HA (green; bottom panels), with GABA expression (inset). Dorsal up, posterior view (left panels); Anterior up, ventral view (right panels). Scale bar: 10 µm

Supplemental Figure 6. Approach to identify NB5-2 Hb+ presynapse neuropil subregions. Neuropil labeled with N-cadherin (white) to define the neuropil border (magenta dotted line; left panel). The neuropil border (magenta) was defined using Imaris 10.0.1 surface tool to find the centroid (cyan dot) of a hemisegment and the center of Idun1-3 presynapse position labeled with Brp staining (green; middle panel). Shown is the Idun2 presynapse neuropil position (N2; yellow dot). The average N2 coordinate location was then found by measuring the dorsal and lateral distance from the centroid (yellow solid line; right panel). The size of the presynapse volume (white box) was determined by the average distance from the centroid +/- 2 standard deviations. Presynapses were simplified using the Imaris 10.0.1 spots tool (grey dots) for quantification. Dorsal up, posterior view. Scale bar: 5 µm.

bioRxiv preprint doi: https://doi.org/10.1101/2024.10.07.616945; this version posted October 7, 2024. The copyright holder for this preprint (which was not certified by peer review) is the author/funder, who has granted bioRxiv a license to display the preprint in perpetuity. It is made available under aCC-BY 4.0 International license.

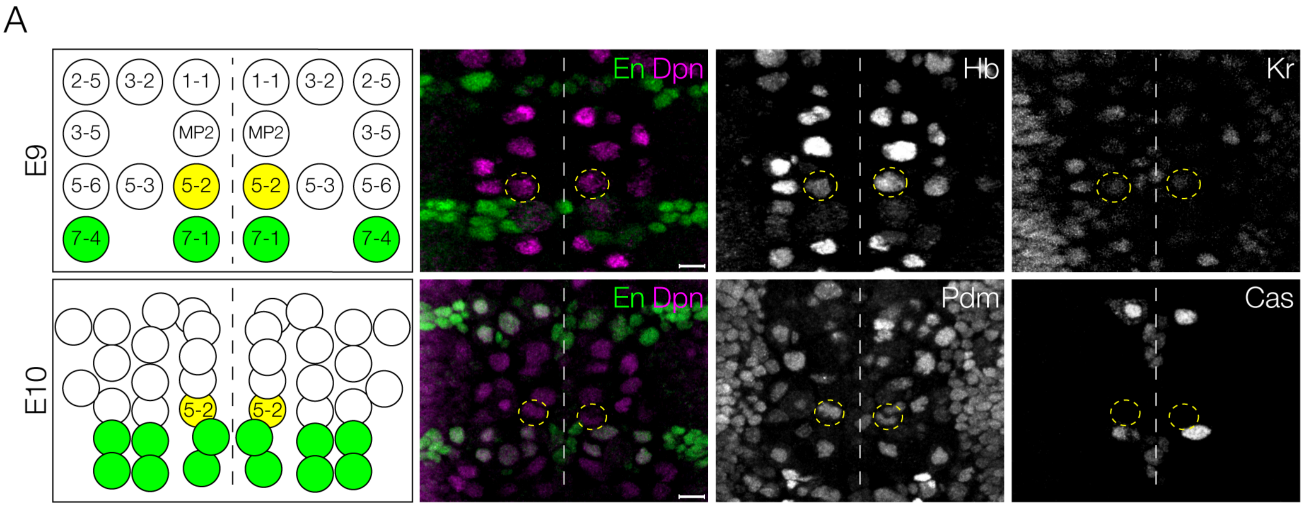

963 Supp Figure 1

bioRxiv preprint doi: https://doi.org/10.1101/2024.10.07.616945; this version posted October 7, 2024. The copyright holder for this preprint (which was not certified by peer review) is the author/funder, who has granted bioRxiv a license to display the preprint in perpetuity. It is made available under aCC-BY 4.0 International license.

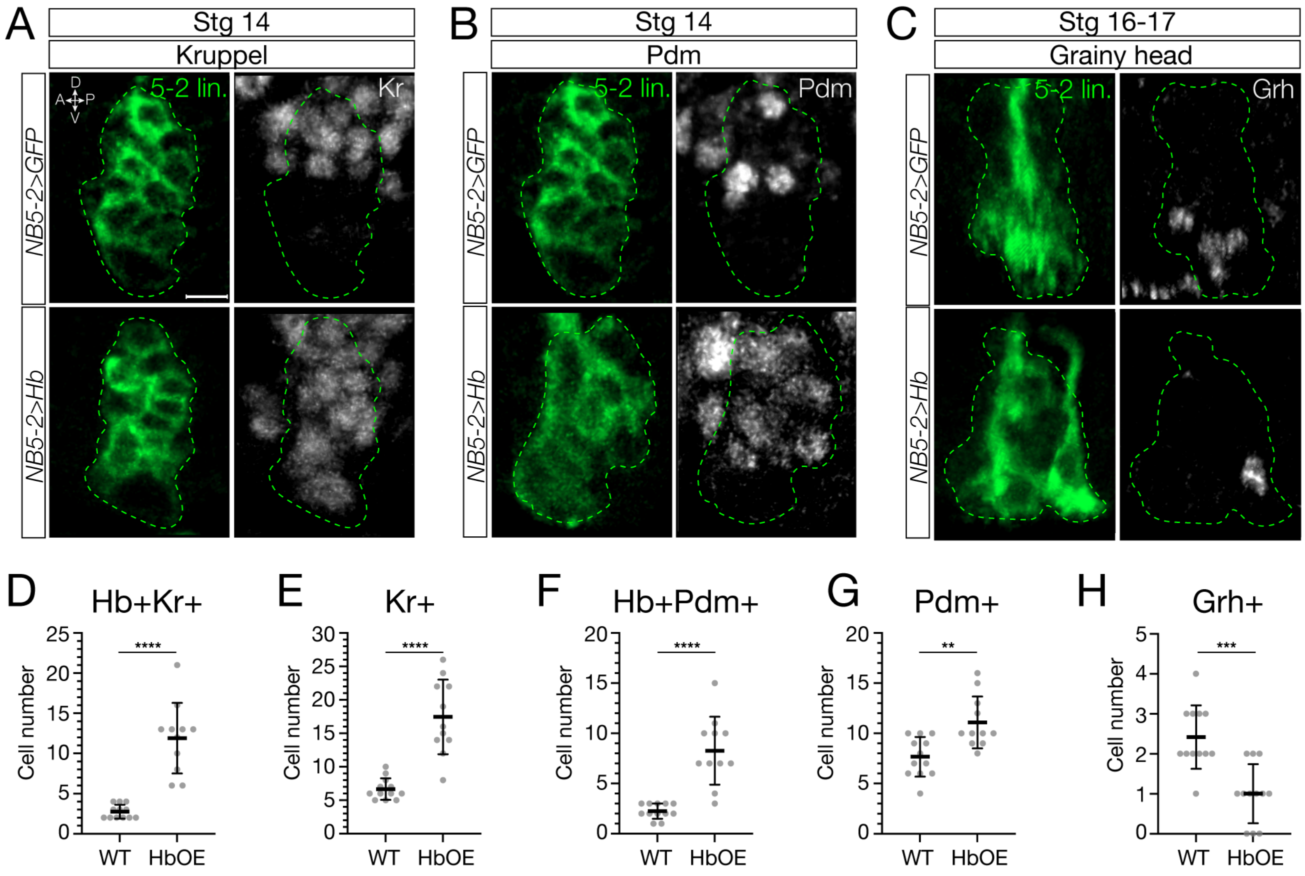

Supp Fig 2

964

bioRxiv preprint doi: https://doi.org/10.1101/2024.10.07.616945; this version posted October 7, 2024. The copyright holder for this preprint (which was not certified by peer review) is the author/funder, who has granted bioRxiv a license to display the preprint in perpetuity. It is made available under aCC-BY 4.0 International license.

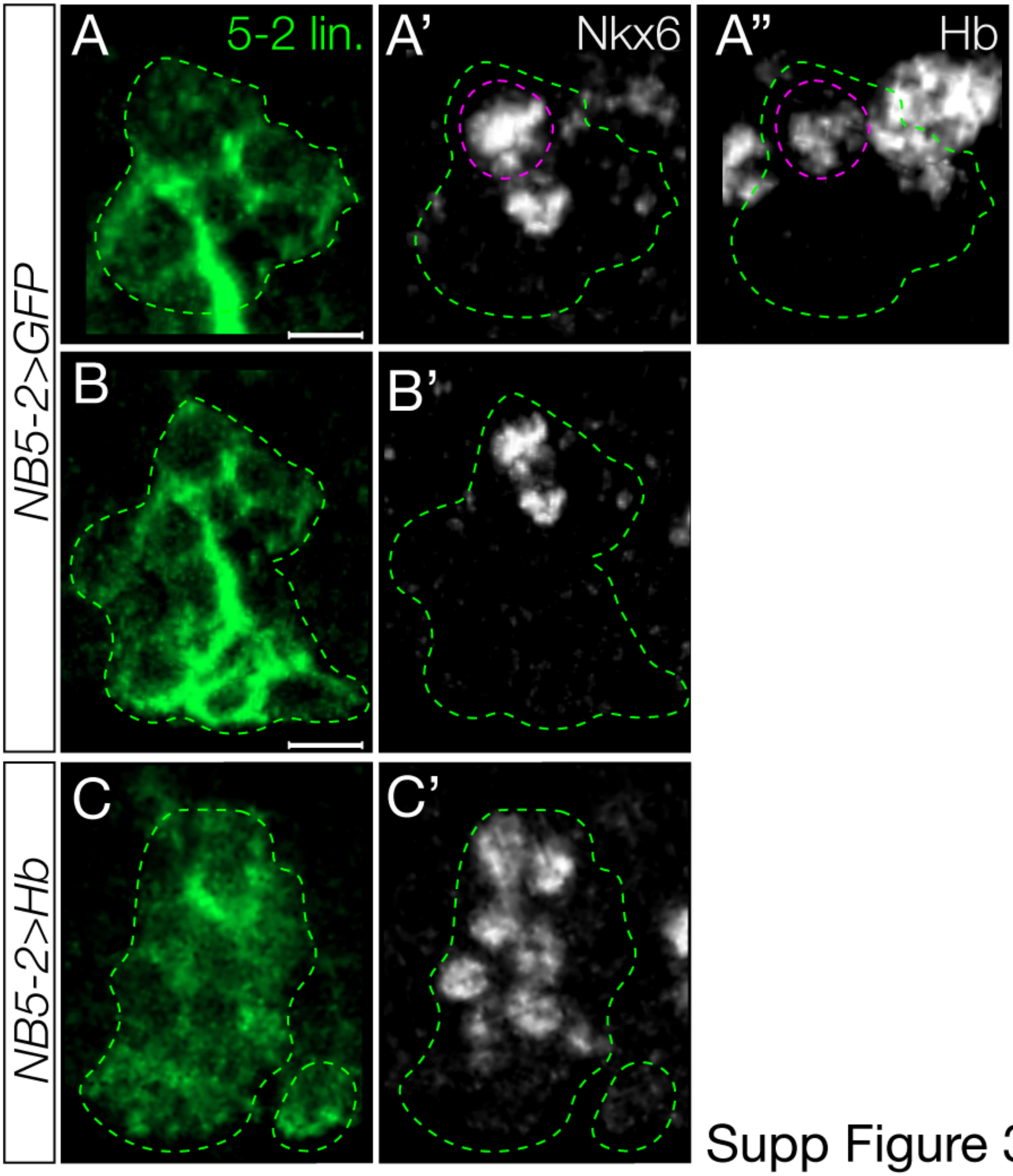

Supp Figure 3

965

bioRxiv preprint doi: https://doi.org/10.1101/2024.10.07.616945; this version posted October 7, 2024. The copyright holder for this preprint (which was not certified by peer review) is the author/funder, who has granted bioRxiv a license to display the preprint in perpetuity. It is made available under aCC-BY 4.0 International license.

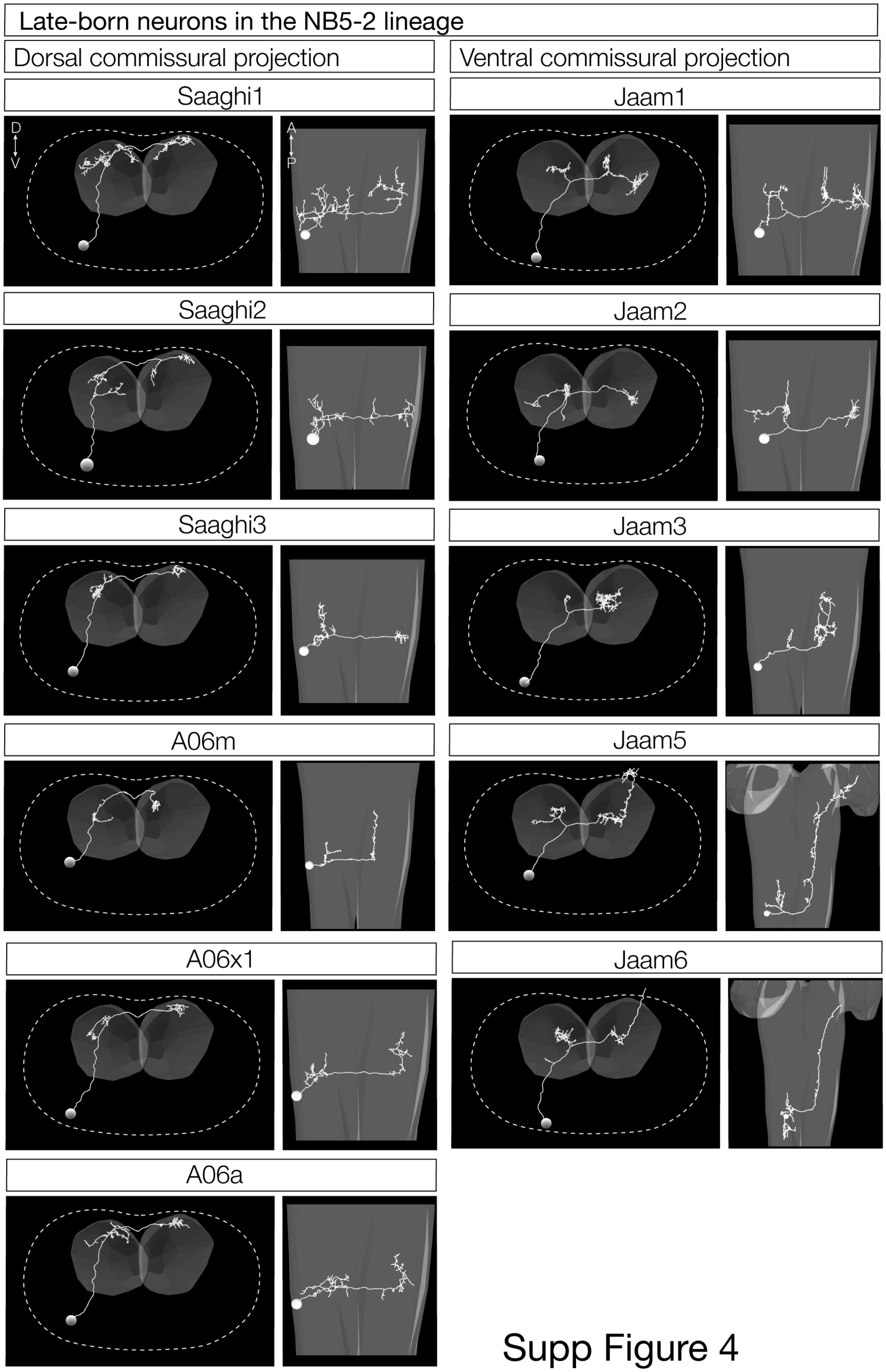

966

Supp Figure 4

bioRxiv preprint doi: https://doi.org/10.1101/2024.10.07.616945; this version posted October 7, 2024. The copyright holder for this preprint (which was not certified by peer review) is the author/funder, who has granted bioRxiv a license to display the preprint in perpetuity. It is made available under aCC-BY 4.0 International license.

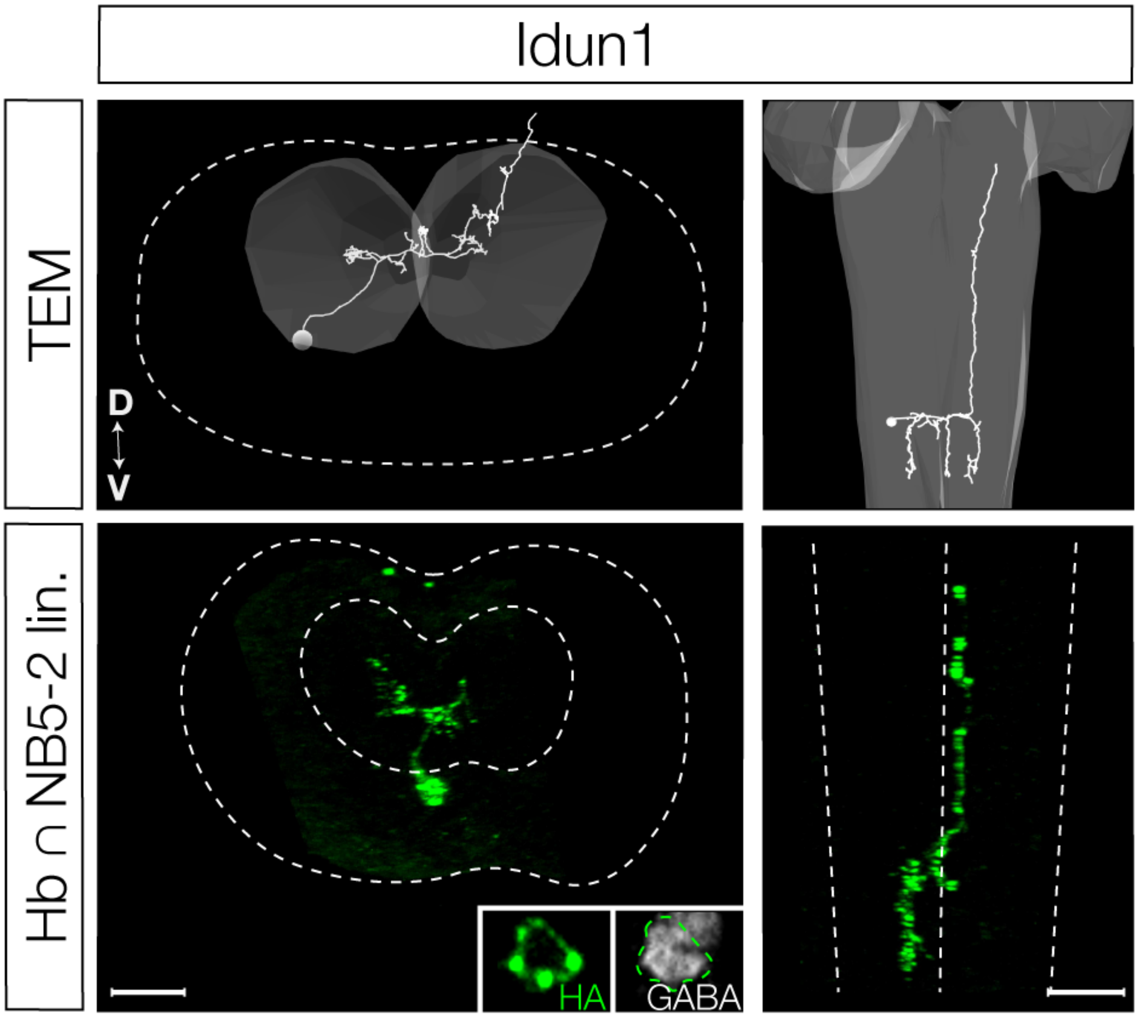

967 Supp Figure 5

bioRxiv preprint doi: https://doi.org/10.1101/2024.10.07.616945; this version posted October 7, 2024. The copyright holder for this preprint (which was not certified by peer review) is the author/funder, who has granted bioRxiv a license to display the preprint in perpetuity. It is made available under aCC-BY 4.0 International license.

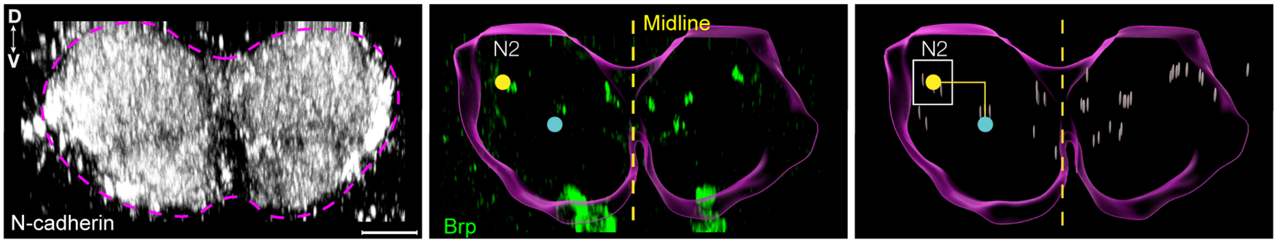

968 Supp Figure 6
